# Online architectural education: Reflections on COVID-19 emergency remote learning in East Africa

**DOI:** 10.1177/20427530221117329

**Published:** 2022-08-18

**Authors:** Mark RO Olweny, Alex Ndibwami, Achilles Ahimbisibwe

**Affiliations:** School of Architecture and the Built Environment, 4547 University of Lincoln, Lincoln, UK; Department of Architecture, College of Science and Technology, 58620 University of Rwanda, Kigali, Rwanda; Faculty of the Built Environment, 108117 Uganda Martyrs University, Nkozi, Uganda

**Keywords:** Architectural education, COVID-19, educational technologies, emergency remote teaching and learning, online education

## Abstract

This paper investigates how students in two schools of architecture in East Africa, engaged with educational activities during the early phase of the COVID-19 lockdown. The COVID-19 lockdown and shift to emergency remote teaching and learning raised a number of questions for architectural education. These relate to access, equity and pedagogical approaches, which emerged through this study. The paper presents the findings of the study carried out in the University of Rwanda, and Uganda Martyrs University, along with the implications of the findings for architectural education. Making use of an online questionnaire distributed via QualtricsXM, the study attracted 70 student participants. The paper concludes with some suggestions for architectural educators as they rethink the embedded pedagogical traditions of architectural education, and how these must adapt for the future in order to cope with future shocks and disruptions.

## Introduction

During the first quarter of 2020, universities across the globe shut their doors, a consequence of health-related directives precipitated by the COVID-19 pandemic. Compelled to move online, universities across the globe took advantage of available communication and information technologies to ensure that teaching, learning and assessment could continue during the emergency lockdown. Initially, it was believed this emergency remote learning was a short term stop-gap measure, with a return to ‘normalcy’ within two to three months. Little did we know that many months and numerous lockdowns after the initial order, many countries still face uncertainty with education having to navigate constantly changing rules and regulations brought on by this situation. Among the many challenges was the question of how to continue architectural educational activities in the absence of the physical design studio, at the core of its signature pedagogy (Shulman, 2005). Further, the underlying presumption of emergency remote teaching and learning was the availability of accessible and reliable information technology. However, what happens when these expectations are not met in reality? This was a scenario faced by educators and students in many parts of the world, more so for architectural education across many countries of the Global South.

The hasty transition to online teaching and learning across the globe created a challenge for educators and students alike, with the pivot to online teaching and learning leaving little time to test the efficacy of e-learning tools or explore benefits of different pedagogical approaches. Faced with this sudden shift in circumstances, we investigated the transition to online teaching and learning in the context of two architecture schools in the East African Community.^1^ The paper presents some of the user experiences that emerged from this transition, invaluable in appreciating the experiences of educators and students during this unprecedented change in circumstances. It investigates how faculty and students continued with their educational activities during the early phase of the COVID-19 lockdown. For architectural education, the move online has forced a rethink of the commonly accepted pedagogical approaches, with the paper concluding with some considerations to aid the development of protocols for future online endeavours.

### Educational technologies in East African higher education

For optimal use of online teaching and learning opportunities, familiarity with the different technologies used to facilitate this mode of education is presumed. In the years prior to the onset of the pandemic, the two universities taking part in this study had installed learning management systems based on the Moodle open-source platform. However, they had not been in mainstream use for regular teaching activities prior to the COVID-19 lockdown. The challenges with uptake of learning management systems were raised by Mabusela and Adams (2017) in South Africa, citing a lack of equipment and staff able to make use of these systems, also found by the International Council for Open and Distance Education (2020). These findings and subsequent recommendations were key to the setting up of National Research and Education Networks (NRENs), which sought to leverage economies of scale to improve the uptake of information technologies across sub-Saharan Africa, and to ensure academic staff received adequate training in the use of information technologies. Organisations such as the Research and Education Network for Uganda (RENU), and the Rwandan Education and Research Network (RwEdNet), have been instrumental in increasing the use of information technologies in universities in the respective countries, driven in part by the popularity of distance learning programmes in the social sciences. Architectural education, for a host of reasons, relied heavily on traditional teaching and learning approaches, in part driven by historic limitations on available technologies, but also due to validation requirements imposed by professional bodies, as highlighted by Tshabalala et al. (2014). For Nsibirano and Kabonesa (2013), the challenge extends to a lack of motivation for staff to make the shift to technology driven educational approaches. For many instructors, the pedagogical approach they adopt is based on their experiences as students. Many existing academics in architectural education have been through educational systems that did not make use of technologies, and the basis for their own teaching approach (Olweny, 2017). The move to emergency remote online learning and teaching presented an additional set of challenges for education, including load shedding,^2^ limited internet access, insufficient workspaces and lack of computer access acknowledged as hindrances to learning (Czerniewicz et al., 2020), however at the beginning of the global transition to online teaching and learning, the extent to which students needed support was not fully recognised. For Olweny et al. (2021), this presumption was a consequence of enduring ontological approaches that have privilege a hegemonic approach to learning and teaching as an ongoing epistemic injustice (Fricker, 1998; Tamale, 2020). These unprecedented changes to education affected faculty and students immensely, and in vastly different ways.

Neither of the schools of architecture included in this study had made extensive use of the Moodle based learning management system prior to the COVID-19 constrained transition, but they had made efforts to advance the use of teaching and learning technologies in their respective architectural programmes. In the architecture school at Uganda Martyrs University from here on, UMU exploring the use of technologies has been underway since 2012 as part of efforts to enhance teaching and learning. This began with the use of the Wiki’s on a MacOSX Server on the local area network as a means to manage teaching material, and to aid the development of independent learning skills in students. The use of Moodle had been tested in 2015; however, network limitations side-lined that project. Since the 2018 migration of the university communication services to Google for Education, the faculty has made use of Google Classroom as part of its teaching and learning strategies. For the architecture school at University of Rwanda (from here on, UR the learning management system has been available since 2013. Efforts had also been made to provide training to enable faculty to make use of the system; however, by the beginning of the lockdown in March 2020, only two instructors had begun the transition. In all, by the time of the lockdown in early 2020, only limited use was being made of Moodle for teaching in the architecture programmes.

For the students, limited availability of IT resources on the university campuses did not appear to affect their use of computers, with virtually all students submitting work that had been developed and meticulously rendered on computers, suggesting that they did have access to good quality computer equipment away from university. University computer facilities were often inadequate or underpowered for the needs of architecture students. Even where facilities were available, at times there was a lack of technical competence to manage these facilities, leaving the computers non-functional for extended periods of time. This was true of the architecture computer suite in UMU which had been set up with brand new iMac workstations in 2015 but was rendered inoperable in 2017 impacting on teaching and learning in the faculty for close to 12 months. Whereas the same could generally be said of computer facilities at UR of particular concern though was a stalled Government programme to supply Positivo laptops to incoming students, which affected students in the architecture programme. Despite this, what it did suggest is that students did have access to and were able to make use of computers beyond the university setting. The decision to move online therefore came as a general acknowledgement of this as well as an understanding that access to equipment would not be a limitation for students.

### Research context and methods

The actual experience of teaching and learning was at the core of the study, looking at courses that moved online, how they were conducted, and how students were engaged on these courses. In UMU, most courses running at the time were moved to online sessions, albeit with a drastically increased contact schedule approximately 50% what it was before the move online, making use of Google Classroom, which had been tried as a means of streamlining student feedback and feedforward. This had been used in conjunction with traditional face-to-face teaching engagements. Consequently, as some students and faculty were already familiar with this system, it was deemed the most appropriate to roll out. However, there was less familiarity with the university's Moodle based learning management system, which had been installed but had not been in wide use. At UR, faculty were compelled to move courses onto the Moodle based learning management system following steps provided by the Centre of Teaching and Learning Enhancement. The goal at the time was not necessarily to complete courses, rather, it was to keep students busy learning on their own (see Appendices A and B for courses moved online). For both schools, it was important to ensure continued and adequate communication between faculty and students, during originally timetabled sessions at the very least.

The paper reports on the findings from a study carried out during the first six months of the COVID-19 lockdown. Undertaken in May and June 2020, the study investigated the experiences of students as part of the move to emergency remote teaching and learning in two schools of architecture in East Africa: UMU, and UR. Both schools of architecture had made significant attempts to continue educational activities remotely rather than shutting down altogether. The two architecture schools are relatively small, having first-year intakes of about 30 students for UMU, and 40 for UR having a student population of 80 and 120 students respectively, giving a potential study population of 200 students.

The study makes use of a questionnaire, incorporating both closed and open-ended questions to gather information on student’s experiences as part of the emergency remote learning. Questions solicited information on study conditions, opinions about experiences, as well as attitudes toward emergency remote learning. Development of the questionnaire went through a series of steps to determine the most appropriate questions to ask, and how to ensure useful information was captured while at the same time ensuring that it was not intrusive and significantly was accessible via different devices, acknowledging that many students access internet content via cell phones rather than laptop or desktop computers. Questions investigated how students carried out their educational activities, the equipment they used, as well as their thoughts on how these experiences impacted their learning. The final set of questions was settled on after a series of consultations with colleagues, and a pilot study, with the questionnaire distributed to graduate students and faculty across East Africa. This helped in fine tuning the language of the questionnaire, and the sequencing of questions. As a result of this process, some questions were changed to ensure they were understood by the target audience, while a few were omitted to ensure that the study could be completed within 10–15 minutes. Ethical approval for the study had been received from the lead authors’ institution prior to the recruitment of participants. Calls to participate were also circulated via social media platforms, Facebook and WhatsApp, to ensure that the questionnaire reached as many potential participants as possible. While the study targeted both faculty and students, for this paper, we report on the findings of the student survey, which yielded 78 responses, representing 45% of enrolled students. Of the students that responded, 56 identified their gender, with 75% identifying as male and 25% as female. There was a good spread of responses from across the different year levels, providing a good indication of experiences across the schools (see Table 1).

**Table 1.** Participants by year levels (Source - NVivo).

Item	UMU	UR	Total
Year 1	12	13	25
Year 2	12	4	16
Year 3	9	5	14
Year 4	3	2	5
Year 5	3	7	10
**Total**	**39**	**31**	**70**

A pertinent question arising from the methodological approach selected, but more significantly from the reality of the context in which internet access is not widespread, is how students were presumed to continue their studies? Across East Africa, government directives were geared at ensuring students were engaged during the lock down. This was through teaching via radio and newspaper for primary school education, and the implementation of ‘zero-rated’ educational internet sites, that ensured free access to secondary school and university sites with designated educational internet domain names (Baike, 2020; Iliza, 2020).^3^ This is interrogated further later in the paper. This did not address the wider challenge of regional disparities in accessing the internet, but created a paradox for the current study, with those unable to participate, an important demographic that could not be contacted due to COVID-19 restrictions in place at the time.

While specific demographic data was not gathered as part of the study, as this was not considered vital at the time of the study, information from the architecture schools provided an idea of the demographics of the students in the two schools. In both cases, students came from diverse backgrounds, and from different regions of the two countries, with their homes located in rural and urban areas alike. Student intakes cut across socio-economic classes, a consequence of deliberate intake criteria for UR. For UMU, an equally diverse population was evident, achieved as a result of intake criteria that acknowledged that the intake criteria based solely on the high school leaving exam was problematic, as highlighted by Liang (2004). A consequence of this approach was that students admitted to public universities often come from a few select schools. Indeed, for one prominent university in the region, of all students admitted to its architecture programme for the 2021/22 academic year, 50% came from a single prestigious school.^4^

The approved questionnaire survey was distributed via QualtricsXM, which allowed automatic reformatting of the questionnaire for different devices: both mobile and fixed, and automatic redirecting of different users to specific parts of the questionnaire. QualtricsXM was also used for initial analysis, making use of its in-built tools which provided basic statistical analysis capabilities. Qualitative analysis of data derived from the open-ended questions was undertaken using the qualitative research analysis software *NVivo*. Analysis of the data made use of constant comparison analysis and classical content analysis as a means to derive meaning from the data, acknowledging the value of triangulation to ensure consistency in the findings (Leech and Onwuegbuzie, 2007). Within *NVivo*, responses were coded into subcategories that enabled a more detailed analysis of the data. An important part of this process was the need to view and evaluate the data from multiple perspectives, a means to ensure comparability, and to verify that categories were meaningful and valid. As such, to ensure validity and trustworthiness in the research findings, it was necessary for different people to evaluate, read and re-reading the findings on separate occasions. This process was undertaken by the three researchers who evaluated and categorised the qualitative data independently. These classifications were then harmonised across a series of sessions, resulting in the final categories presented here (See Appendix C). Through this process, it is possible to ensure the trustworthiness of the interpretations, and that they meet the trustworthiness criteria as presented by Nowell et al. (2017). Significant also is the notion of ‘rightness’, that requires an acknowledgement that there are no universal truths. which often negate the value of ‘the other’. For Goodman and Elgin (1988), rightness is multi-dimensional, broader in scope and more complex than the truth. It is therefore important that statements from respondents were reproduced verbatim, a means of transmitting to the reader the voices of the participants in the study.

### Venturing into online architectural education

In analysing the findings of the study, two areas stood out as being particularly significant and thus form the basis for the ensuing discussions in this section. The first relates to conditions leading into and under which students had to work. This includes preparation, expectation and anxieties related to the unexpected situation, along with the realities associated with the student’s domestic situations. The second relates to teaching and learning, and student’s engagements in these activities.

### Preparation, expectations and domestic realities

The unprecedented shift to emergency remote teaching and learning during the first half of 2020 left little time to ponder the direction this educational experience would take. For architectural education, any move from the traditional face-to-face on-site studio-based approach has always been contentious (Fleischmann, 2020). The design studio, the long-standing and revered signature pedagogy of architectural education is largely viewed as indispensable. However, the COVID-19 pandemic brought design studio activities to a standstill, compelled architecture schools to abandon this tried and tested approach, and to seek alternatives. The initial lockdown had been presented as precautionary given there were no known COVID-19 cases in either Rwanda or Uganda at the time. The initial closure, mandated by the governments of Rwanda and Uganda, was to be for a period of 30 days. During this period, universities and other institutions of higher learning were to develop strategies by which they would carry on educational activities online. To enable the transition to emergency remote teaching and learning, universities and schools of architecture scrambled to provide guidance to faculty and students to ensure educational activities could migrate online. Guidance ranged from simple notifications of the courses students would undertake and the online platforms that would be used, through to, procedures and conduct of online activities. The value of this guidance becomes evident when we look at the familiarity of students with educational technologies. Approximately 80% of respondents indicated they had limited experience with online educational systems: 42% (39) of students indicated that they did not have prior experience with online learning, with 38% (35) having one year or less (see Table 2). While the findings could indicate that students in the first year, may have been unfamiliar with the educational technologies, these responses to raise a significant question of when and how students are introduced to these technologies when they enrol?

**Table 2.** Experience with educational technologies (Source - QualtricsXM).

Prior experience with e-Learning	Count	Percent, %
None	*39*	*41.94*
Less than 1 year	*35*	*37.63*
1 - 2 years	9	9.68
3 - 4 years	3	3.23
More than 5 years	1	1.08
Missing	6	6.45
Total	93	100.00

For many students, this meant seeking clarity on how to access their respective learning management system and securing the necessary log in credentials. As one student noted: “*It gave me a start to the understanding of online teaching, also what to expect and what not*” (#58). Responses indicated that guidance received by students had mostly come from academic faculty and was not general guidance given by the universities. This would suggest that special consideration may have been made for the needs of architectural education. The guidance provided support in a variety of areas, from access and engagement protocols to the nature of activities that would be carried out, along with general help and reassurance designed to motivate students as they embarked on this unfamiliar learning environment (see Table 3).

**Table 3.** Guidance provided (Source - NVivo).

Guidance	Mentions	Sample response
Provided motivation to engage/Continue studying	18	“It gave me a hint of what to expect, and how to maximise the experience.” (#106)
Clarifying communication approach/Channels	13	“We had already established a google classroom that was used to share progress, feedback and precedents with the class and course coordinators.” (#36)
Help in navigating online platforms	9	“It helped me understand that help was there when I needed it. All I had to do was ask or better yet participate in the activities offered.” (#69)
Provided a basis for activities	5	“helped me to go forward even during this pandemic and I experienced to work more activities.” (#74)
Not very useful	3	“Not very much only an email was sent and materials found on the platform are not well elaborated.” (#85)

While overall there was an acceptance that the guidance given was useful, for some students, it was not very useful, and in some regards, frustrating given the haste in which things unfolded: “*We were only told that we would right before quarantine started but had no time to mentally and financially prepare for it. It is in most cases inconveniencing but at the end of the day its meant for our own good leaving as no choice but to try. Online learning is not as efficient as physically attending class and hence leads to lower performance*” (#57)). The trepidations emerged from all levels of the student body and could be traced back to the domestic situations in which students returned to as part of the lockdown.

**Figure 1.**
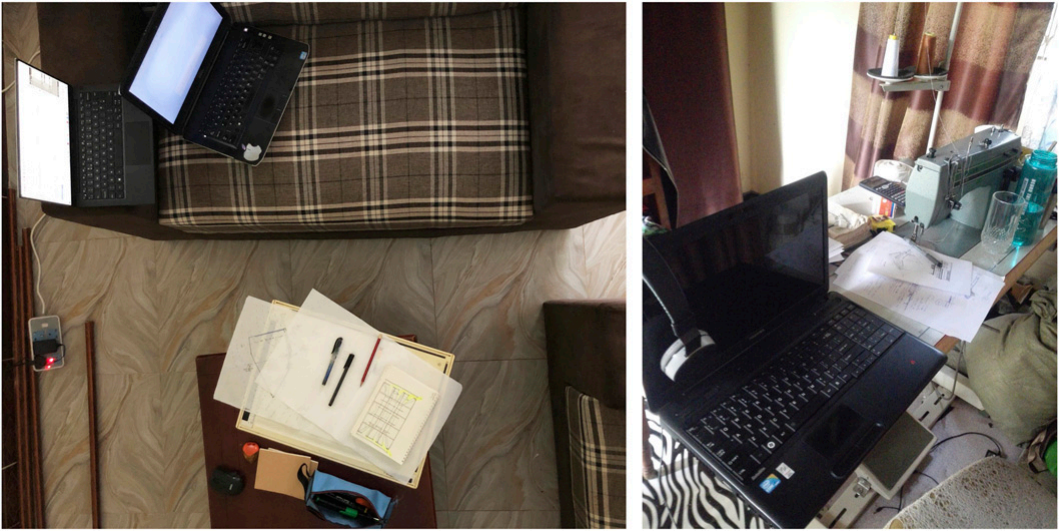
Home Study Spaces (Source - Questionnaire).

The pandemic removed students from the design studio, placing them in their home environments, which was disruptive at many levels. At a general level, the education system in Rwanda and Uganda is based on a boarding school system. From Senior 1 (Year 8, through to the high school leaving exam at Senior 6 (Year 13), and through to university, virtually all students are resident at their schools. Consequently, the move back home was a challenge because it placed them in a situation that was unfamiliar. Many students share bedrooms with siblings, having very limited space to work, with numerous “*DISTRACTIONS*” (#14), and conditions that were regarded as not being suitable for learning: “… the environment at home is not conducive for learning” (#11). Having a space to concentrate and lay out work was difficult for some students, citing challenges related to shared spaces with limited space to spread out work: “*It’s hard to draw at all, since we need specific tables, instruments and collaboration with lecturer, but we’re trying drawing from sketches and on small papers like A4 papers rather than A3*” (#25). This was a common challenge for students, as presented in the images submitted (See Figure 1)

Linked to these home environments and emerging from the responses were issues related to student’s mental wellbeing. A few students indicated that the lockdown increased their anxiety: “*Working alone is also challenging and requires a lot of courage and morale which isn’t mutual to everyone at times*” (#6); “*Mentally it’s challenging, as I feel alone and i end up having anxiety attacks*” (#17)); “… here you work alone which is not as motivating” (#57). Migration to online learning, implemented rapidly and little time to prepare may have exacerbated challenges for students, enough for them to raise this as an issue, which is often hidden in the context of sub-Saharan Africa, although anecdotal evidence suggests it is a growing concern, more so in professions education (Bantjes et al., 2020; January et al., 2018; Ovuga et al., 2006). Much of this could be related to the blurring of personal and professional (student) lives, occurring in ways students were not familiar with.

### Teaching and learning activities

Both schools of architecture were compelled to use their respective Moodle based learning management systems as the main interface between faculty and students. This was tied to the ‘zero-rated’ educational sites, designed to ensure continued access to university services by removing the cost of data associated with accessing this specific internet portal. The challenge of course was that this related only to specific university sites, and curiously did not include access to university libraries. While this zero-rating was welcomed, it did raise a question of how students learn in higher education. Free access only to the learning management system suggested that all learning material would be made available on this portal, which for university students, is certainly not possible. This reality was not lost on students, as noted in Table 4. Significantly, load shedding was not mentioned as a challenge, despite its potential impact on engagement.

**Table 4.** Technology challenges (Source - NVivo).

Challenge	Sample response
*Internet*	*“Lack of computers and luck enternet connection.” (#73)*
	*“There is an unreliable internet that affects participation in online education.” (#20)*
*Access to Information*	“The major problem we face is inaccessibility to materials, internet and proper tools or gadgets for the online sessions.” (#21)
	*“Some online resources were provided by the school like access to jstor, but now its requires money.” (#61)*
*Cost* *Computer Hardware*	*“The cost of internet data” (#8)* *“The challenges now is materials like computers which is my most discouraging challenge” (#90)*

The ubiquity of mobile devices as the key means of accessing the internet inevitably meant that it was a likely determinant in how communication was undertaken between students and faculty. The rapidity of the transition to online activities compelled students and faculty to make use of any available, reliable and effective systems and readily accessible beyond the confines of the university setting. The absence of face-to-face interaction brought social media into the fore, a means by which faculty could connect with their students and vice versa. For students, for whom social media is a lifeline, the true value of social media was made apparent during the lockdown.

Social media platforms including Instagram, Twitter, WhatsApp and YouTube emerged as important sources of information, and means of communication. The importance of students as a demographic saw telecom companies in both countries developing new internet packages directed specifically at students as the largest social media users. Even before the lockdown, and due to its large subscription base, the ability to send and receive large files, and free telephony, WhatsApp was the go-to social media platform, and the primary means of communication among students. For many students, this soon became the platform by which they could communicate with faculty: “*Through WhatsApp groups (where a lecturer is among the members), all classmates are able to get updates about the course, as well as expressing our opinions to the lecturer*” (#51); “*Perhaps in my class, we made a WhatsApp group where we can review each other’s work and later give feedback*” (#55); “*But we mainly use WhatsApp to discuss the difference assignment and give each other feedbacks*” (#12); “*We use WhatsApp group for daily communication and discussions*” (#99). While WhatsApp was a key means for general communication with peer groups, for communication with faculty, email and Google Classroom were predominant. Submission of work was also through these platforms See Table 5). Video conferencing, making use of Zoom or Google Meets, was also seen.

**Table 5.** Modes of communication (Source - QualtricsXM).

Response	Communicating with instructors	Communicating with peers	Submission of work	Total
Email	26	5	29	60
Dropbox	—	—	12	12
WhatsApp	6	25	5	36
Learning management system	2	—	12	14
Video conferencing	9	6	—	15
Phone call	1	6	—	7
Google classroom	24	13	29	66
Total	68	55	87	210

The prominence of Google Classroom is a reflection of the familiarity of faculty and students in UMU with this particular platform, which had been in use for at least a year, and was easily accessible on and off campus. An important element of Google Classroom was that in conjunction with a drawing tablet, it allowed faculty to mark-up student submissions, and automatically save marked up work for instant access by students. At the UR, one instructor made use of Dropbox to allow students to submit work, particularly useful for large file types. The choice of the various modes of communication suggests these were selected based on faculty preferences (Google Classroom and DropBox), or negotiated between students and faculty (WhatApp). This does highlight the challenges thrust upon higher education by the move to emergency remote teaching and learning, indicating a high level of adaptability by faculty and students in attempts to continue educational activities.

The chosen platforms were also determined by the mode of access to internet services. When physically present on campus, students generally had access to limited computer resources and unlimited internet. The latter is significant as access to the internet across the region is still fairly limited outside businesses and educational organisations. Few households have wired telephony or internet, making use of cell phones or sim enabled modems through which desktop or laptop computers could be tethered, generally paid for via ‘airtime’ or ‘data bundles’. Unsurprisingly, access to internet services was the most reported challenge for students after the move to emergency remote teaching and learning, given it entailed additional expenses for data and required a computer or laptop that was able to tether to a cell phone or wireless modem to access the internet, which wasn’t always available. This was a determining factor in the choice of communication platform, and the preference for asynchronous activities modes over synchronous engagements (Video Conferencing and Phone Calls).

Attempts to carry on synchronous activities online notwithstanding, it proved difficult to sustain activities as there was increasing level of miscommunication reported by students, more so for design related activities, building frustrations among students: *“It affected my learning negatively since sometimes I need physical contact with supervisor so that we can do some sketches together”* (#50). This highlighted another challenge that only came to light later, the difficulty in giving and receiving feedback, more so as communication of ideas in architectural education has traditionally relied on not only written and graphic communication, but also on the emotional signals transmitted and received (Melles, 2008). The absence of this element of communication was highlighted by the students: “*Generally, I have realised a need to there’s a need to be more deliberate in order to learn especially from fellow students*” (#101). These and other such statements highlight the importance of nonverbal cues as part of communication geared to aid learning, which was absent when engaging remotely, more so in asynchronous engagements.

The lack of computer hardware proved to be a barrier for participation in online education, with several students indicating that they did not have computers at home: “*Some of us don’t have computers*” (#12); *“the challenges now is materials like computers which is my most discouraging challenge”* (#90). In some cases, even having an available computer did not guarantee access to the internet as many old model laptops and desktops lacked updated software with the necessary security updates required for access to many internet sites. Further, some students indicated that a lack of electricity made it difficult to have the needed access at times when they needed to, or even the case where students lived in remote towns and villages where internet services were not available: “*Say some students live in areas where there us limited internet access*” (#53); “*This is really challenging period where some of us in our place of residence there is no power. I have to travel to reach the place where there is power to work on my project and Thesis Booklet*” (#83). In addition to limitations on hardware, there were also challenges with availability of software. While it is now commonplace to have a basic suite of productivity software available on the various computer devices, such as Microsoft Office (Excel, PowerPoint, and Word), Apple iWork (Keynote, Numbers and Pages), and now Google (Docs, Sheets and Slides), this is not the case for specialised software for image processing (e.g. Adobe Illustrator, Photoshop) and computer aided design and drafting. (e.g. Archicad, AutoCAD and Vectorworks). This is where the value of on campus facilities have been important, as they gave students access to professional software they would otherwise not have had. The inability to access this software during the lockdown was difficult, compelling students to make use of alternative means of engaging with their project work.

Notwithstanding all the challenges raised in the move to emergency remote learning and the loss of the physical studio learning space, it was heartening to note that they did endeavour to keep in touch with their peers (both socially and academically). Students made use of WhatsApp for this purpose, with one group of students (on their own initiative) setting up a buddy system, a means to support each other and to keep themselves motivated during the lockdown. This WhatsApp group was used to share ideas and to give comments on work being undertaken: “*We as a class have set up a buddy system where one person is accountable to 2 people so that we review each others work before being sent to Google Classroom*” (#44); “*I’m engaged with a few peers. Reliable peers those that can text back real quick. Plus those whose critique you trust*” (#27). The buddy system did highlight how communication could be continued outside of the physical studio space, although the serendipitous encounters of the physical space are absent. Students were quick to recognise the opportunity this created for collaboration and exploration beyond the confines of the physical studios, schools, universities, and counties: “*Collaboration with a wide variety of students both nationally and internationally*” (#20); “*If we are not all in the same physical location, we should embrace this and have classes delivered by instructors all over the world.*” (#56). Such activities are possible with adequate preparation and coordination, as had been explored by Slee et al. (2016) as part of what they described as a ‘Cyber-Studio’.

## Discussion

The global lockdown in 2020 precipitated by the COVID-19 pandemic raised numerous questions for universities across the globe, more so for architectural education in Rwanda and Uganda which up till then had not strayed too far from the tried and tested approaches evident when architectural education transitions into the university setting a century earlier. This study has highlighted how two universities in East Africa sought to transition into emergency online teaching and learning, a process that raised various challenges along the way, some that could have been anticipated, but others that were discovered along the way. It was this transition that caused faculty and students to seek means to continue teaching and learning. The necessary adaptations to activities, having to build capacity in the use of unfamiliar software and disparate access to computers and internet services. This raised significant challenges for all concerned, while at the same time highlighting opportunities for architectural education to transform in the face of a changed educational landscape, foregrounding queries of the presumed permanence of traditional studio pedagogy. Indeed, as is posited by Karen Lutsky, “… if the space of an office is also changing, maybe we need to be teaching these skills differently anyway” (as quoted in Brey, 2020). Bearing this in mind, it is essential to reflect on the outcomes of the current study, and the potential lessons that could be carried forward.

**Figure 2.**
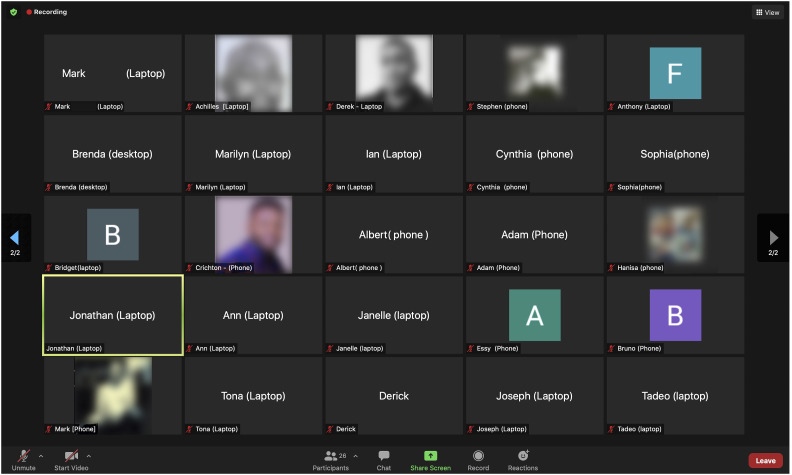
Zoom Class with Devices Indicated (Source - Authors).

During the early phase of the transition to online teaching and learning, there were fears that faculty would be unable or unwilling to participate, a consequence of what is often perceived as a digital divide between students as digital natives and faculty as digital novices, as presented by Prensky (2001). However, it was evident that there were enthusiastic efforts to move activities online. In this case, the apparent digital divide proved to be somewhat of a myth, with evidence that faculty were able to take on different technologies to continue their teaching, and in some cases adapting to make use of different platforms to ensure they were able to deliver their courses. This ability to learn and adapt emerged as a manifestation of a reality of architectural education which involves concurrently ‘learning about’, ‘learning to do’, and ‘learning to be’ (Olweny, 2017). Adapting architectural education to limits of the available technologies became a key factor in this transition. This can be viewed in the context of prior persistent attempts to discourage use of cell phones and social media in education, technologies regarded as disruptive to higher education, but over time have become an invaluable tool in education (El Bialy and Jalali, 2015; Kirschner and Karpinski, 2010; Morkel, 2011; Wanner et al., 2019). It is not unusual to have more than 50% of students logging on via their mobile phones (See Figure 2). It is therefore important that for the set up and design of any online teaching and learning activities that access conditions are kept in mind, even for graphics-based degree programmes such as architecture. Connecting via a mobile device does have its benefits, as it is somewhat insulated from intermittent power cuts (Should the device batteries hold up).

Access challenges also extended to limitations to the availability of essential software. The high cost of essential software made available by universities on campus, was suddenly not available to students. Further, while universities in other parts of the world can dole out software licences to allow students and educators to work from home, this is not feasible for many institutions in the global South. This is a challenge that is difficult to rectify, more so as many companies move toward subscription-based software creating a significant barrier to computer-based teaching and learning across the global South. For universities faced with other more pressing expenses, it is difficult to justify recurring expenditure on ever-increasing annual licensing fees for access to software, a marked change from the stand-alone licences. The same is true for access to eBooks and electronic journals. This in itself highlights the ‘access paradox’ for higher education (Mania et al., 2017), reinforcing the evident disparities among the student population. Even though open-source software options are available as proposed by Van Reijswoud and Mulo (2006), this is not a viable option for architectural education for which specialised software is often required.

The experiences of teaching and learning online demanded a high level of flexibility from faculty and students. Despite the best intention to continue educational activities online, the presumption that students had access to available equipment, and that funnelling content via an e-platform would fulfil the initial objective was not fully achieved. For architectural education, use of a wide range of sources and applications is necessary, as is the need for synchronous feedback, more so for students still in the early stages in the development of architectural values. The use of WhatsApp for instance is an example, where out of necessity, faculty had to go where the students were to keep in touch with their students, a confirmation of an approach described by El Bialy and Jalali (2015), as an essential means to keep in touch with students.

These experiences and engagements begin to touch on issues of access and equity. With students forced to continue their studies at home away from the design studio often presented as the core of architectural education, the added support was a means to compensate for this perceived loss. The loss of the physical space of the studio as part of the emergency remote teaching and learning highlighted the value of this space as more than just a teaching space, but as a key social space crucial in the socialisation of students into the profession of architecture, supporting the findings presented by Tumusiime (2013). For the two schools in this study the studio acted as an equaliser, ensuring all students, regardless of their socio-economic background, were provided with the basic space and equipment to enable them to take on studies in architecture (Olweny et al., 2021). The absence of such spaces in their home environments was certainly a challenge, although the solutions that emerged, using different communication technologies indicated that it was possible to recreate to a degree some of the vital aspects of the design studio and moving towards enabling participation in educational activities even with limited means. What was difficult to replicate was the ability to draw or sketch together as explored by Slee et al. (2016). Regardless, the ability to use existing communication platforms to send and receive files for review did prove useful, more so as this could be done via the zero-rated e-learning sites. The solutions settled on providing an indication that some of the values embedded in architectural education, incorporating aspects of negotiation, exploration, application, and reflection emerged in seeking solutions for emergency remote learning. What the information and communication technologies did provide, was a degree of flexibility in the way teaching and learning could carry on under these circumstances, and in spite of the challenges and limitations both expected, and unexpected.

## Conclusion

There is no doubt that the COVID-19 pandemic and the subsequent shift to emergency online teaching and learning have been disruptive to higher education across the globe. When students were sent home in response to health-related directives, the challenge of moving education online was substantial, more so for programmes such as architecture which do rely on face-to-face interactions as part of their pedagogical approach. Despite the best intentions of carrying on with as little change to schedules, it was soon evident that it was necessary to work toward a revised conceptualisation of how these activities could be undertaken. The experiences during much of 2020 do compel architectural educators to rethink the long-standing traditions at the heart of architectural education. What has been brought into focus is the value of studio-based pedagogy, in particular how it could adapt to changing circumstances. This goes against claims that present architectural education as having a prescribed pedagogical approach which cannot be adapted to changing circumstances, including developments in information and communication technologies. Indeed, as part of the pilot phase of the study, it was revealed that many architecture schools across the East Africa suspended all teaching and learning activities rather than moving them online, in part a consequence of the limited engagement with information and communication technologies as part of teaching and learning.

As universities begin the task of resuming activities, in some cases after a prolonged shutdown, what lessons can we carry forward to ensure that any future shutdowns are not as disruptive to architectural education? This brings forth additional questions of the future state of the design studio. Information and communication technologies across many university campuses across the global South are rudimentary at best, with internet connectivity notoriously unreliable, and with insufficient bandwidth to support large scale synchronous activities, or to accommodate transfer of large files. Efforts are needed to upgrade university systems to ensure they are adequate for educational purposes. Consideration needs to be made for students who do not have ready access to internet services or computers that can access e-learning services. While organisations such as RENU and RwEdNet have worked to provide a minimum level of service for universities, their efforts are hampered by a limited ability to ensure effective access across universities, let alone to remote locations.

Acknowledging that the preparation and delivery of online courses requires intensive and extensive planning, well-catalogued and available teaching material and most important technological support, the experiences of 2020 and 2021 have revealed the areas that can easily be adapted to suit online learning, as well as those that require additional consideration. Certainly, it is essential that all faculty and students are given appropriate training to ensure they can make use of information and communication resources as part of their teaching and learning. Reflecting on the student experiences, it is also necessary to provide students with teaching and learning roadmaps, with goals and targets to help them keep track of teaching activities and learning engagements. Such explicit guidance is essential to prevent stress and mental anguish for students and academic faculty alike. It is also important to assist students in setting up peer learning and collaborative opportunities. While students may know each other socially, this engagement may not always be useful in the educational environment. Students need to be assisted in developing appropriate skills in understanding how to deal with the overlap between personal and academic lives, more so as they can become blurred with online teaching and learning.

It is certainly the case that the early and enthusiastic shift to emergency remote teaching and learning was a result of a belief that this was possible and could be easily effected. While this was achieved, the findings of the study suggest some fundamental challenges emerged at the various levels, for the institutions, faculty and students. While there was significant will to engage with the alternative educational approach presented by the COVID-19 pandemic, and subsequent global lockdown, the outcome could be a significant improvement in how the two schools of architecture approach their educational activities in the long term. This may well be the catalyst that triggers a much-needed rethink and update to architectural education to make better use of information and communication technologies.

## Notes

1. Incorporating Burundi, Kenya, Rwanda, South Sudan, Tanzania and Uganda. There are 11 recognised architecture schools spread across Kenya, Rwanda, Tanzania and Uganda

2. The deliberate shutdown of electric power to manage strains on system capacity.

3. Internet access across Rwanda and Uganda is generally via wireless technologies, and mobile devices. Cabled internet is generally only available in the major urban centres, and generally only for businesses and commercial entities. Wireless modems and cell phones are the predominant means of accessing the internet, which is priced per MB and is not unlimited.

4. As a public university this information is published on their website.

## Appendix A Courses Undertaken Online (UMU)

**Table:**

Item	Some student engagement	No student engagement
Year 1	ENDS-1222 Culture, Climate and Settlements II ENDS-1232 Design Fundamentals II ENDS-1241 Design and Construction Technologies I	ENDS-1252 Natural and Built Environment Systems II LIT-1201 English Literature and Composition
Year 2	ENDS-2212 Buildings and the Environment (Design Studio) ENDS-2243 Design and Construction Technologies III ENDS-2253 Contemporary Landscape Architecture theory ENDS-2505 Special Topics in Design II	PEF-2201 Ethics in Focus
Year 3	ENDS-3271 Architecture Design Project (Design Studio) ENDS-3245 Design and Construction Technologies V ENDS-3205 Special Topics in Design III ENDS-3601 Advanced Studies in Design	BET-3201 Business Ethics
Year 4	ARCH 6541 Building Modelling and Simulation	
Year 5	ARCH-7103 Architecture Studio C (studio)	

## Appendix B Courses Undertaken Online (UR)

**Table:**

Item	Some student engagement	No student engagement
Year 1	ARC1261 Basic Design II ARC1262 History of Architecture II ARC1264 Visual studies II	ARC1263 Architectural Mathematics ARC1265 Physical Environment (Geology & Ecology) EGP1111 English for General Purposes
Year 2	ARC2261 Architectural Design II ARC2262 Architectural Theory I ARC2263 Building Environmental science II: Thermal & Acoustics	EAP2112 English for Academic Purposes ARC2264 Intermediate Digital Representation ARC2265 Building Technology I: Materials & Construction
Year 3	ARC3262 Architectural Theory III ARC3264 Human Settlement	ARC3261 Architectural Design IV ARC3263 Structures II ARC3265 Building Technology III: Building Systems
Year 4	ARC4261 Architectural Design VI ARC4263 Architectural Research Methodologies	ARC4262 Architectural Practice & Entrepreneurship II ARC4263 Architectural Research Methodologies ARC4264 Urban Design EMV3261 Project Management (Elective)
Year 5	ARC5261 Thesis 2.1 Conceptual Design and Resolution ARC5262 Thesis 2.2 Project Representation ARC5263 Thesis 2.3 Design Process Development	

## Appendix C Themes Emerging from the Questionnaire

**Table:**

	Q2.6 - Usefulness of guidance for remote learning	Q2.7 - How are you continuing studio sessions under the social distancing and online engagements?	Q2.9 - Comment on how this engagement has affected your learning?	Q2.11 - How do you receive feedback for the work you are undertaking?	Q2.12 - How have you engaged with your peers during this period?	Q2.13 - What challenges are presented for architectural education by the shift to online education?	Q2.14 - What opportunities are presented for architectural education by the shift to online education?
**1**	Provided motivation to engage/Continue studying (19)	Making use of LMS and E-resources (17)	Negatively affected performance _ less effective (11)	Email (27)	Using social media (WhatsApp) (21)	Limitations of infrastructure (Computers_Power_Internet) (29)	Explore alternative approaches to working (12)
**2**	Clarifying communication approach/Communication channels (13)	Has been difficult (14)	Don’t get to engage with my peers and instructors (9)	Google classroom (24)	Through Google classroom (12)	Difficult to replicate studio environment (17)	Building discipline/self discovery/Confidence/Effectiveness (11)
**3**	Help in navigating online platforms (9)	Ensuring ongoing feedback (13)	Neutral (6)	Video conferencing (8)	Not undertaken (8)	Reviews are difficult _ receiving feedback is difficult (8)	Learning new skills (9)
**4**	Provided a basis for communication (5)	Using email & social media to engage with peers and instructors (8)	Difficult due to lack of faciltities (5)	None (6)	Peer to peer buddy system (7)	Difficult home environments (8)	No opportunities (9)
**5**	No guidance (4)	No studio activity (7)	Communication is difficult (5)	WhatsApp (5)	Not well established or difficult (7)	Can’t build models (6)	More time for research (7)
**6**	Not very useful (3)	Formal online sessions (6)	Mental anguish (3)	Directed to online resources (3)	Video call (Zoom or Facetime) (5)	Lack of clear direction _ Wasted time _ Poor time management (5)	Wider collaborative networks (5)
**7**		Need to be adaptable or Improvise (4)	Keeps me engaged (3)	LMS _E-learning platform (2)	Telephone calls (4)	Limited access to learning resources _ difficulty accessing information (4)	Cost effective/Better access to resources (4)
**8**		Largely self directed learning (3)	I Am more effective and efficient (3)	Face-to-face (1)	Email (4)	Absence of face-to-face interaction (2)	Increased motivation for work/less competition (4)
**9**		Using time to develop skills (2)	No longer learning (3)			Mentally challenging (2)	No anxiety (1)
**10**			Needed to adapt to a different process (3)				Provision of explicit feedback (1)
**11**			Costly (1)				
**12**			Limited access to reference material (1)				
**13**			Not affected (1)				
**14**			Feedback and communication is clearer (1)				
**15**			Appreciate the new learning experience (1)				
	**n = 54**	**n = 57**	**n = 65**	**n = 65**	**n = 63**	**n = 55**	**n = 53**

## References

[bibr1-20427530221117329] Baike P (2020) Students Embrace E-Learning With MTN Zero. Available at: observer.ug/education/65158-students-embrace-e-learning-with-mtn-zero (accessed on 20 August).

[bibr2-20427530221117329] Bantjes J Saal W Lochner C, et al. (2020) Inequality and mental healthcare utilisation among first-year university students in South Africa. International Journal of Mental Health Systems 14(1): 1–11. 31998406 10.1186/s13033-020-0339-y PMC6982378

[bibr3-20427530221117329] Brey J (2020) Teaching and Learning Online, by Force. Available at: landscapearchitecturemagazine.org/2020/03/19/teaching-and-learning-online-by-force/(accessed on 20 August).

[bibr4-20427530221117329] Czerniewicz L Agherdien N Badenhorst J, et al. (2020) A wake-up call: Equity, inequality and Covid-19 emergency remote teaching and learning. Postdigital Science and Education 2: 946–967. DOI: 10.1007/s42438-020-00187-4. PMC7509221 40477091

[bibr5-20427530221117329] El Bialy S Jalali A (2015) Go where the students are: A comparison of the use of social networking sites between medical students and medical educators. JMIR Medical Education 1(2): e7. 27731847 10.2196/mededu.4908 PMC5041347

[bibr6-20427530221117329] Fleischmann K (2020) Online design education: Searching for a middle ground. Arts and Humanities in Higher Education 19(1): 36–57.

[bibr7-20427530221117329] Fricker M (1998) Rational authority and social power: Towards a truly social epistemology. Proceedings of the Aristotelian Society New Series 98: 159–177.

[bibr8-20427530221117329] Goodman N Elgin CZ (1988) Reconceptions in Philosophy and Other Arts and Sciences. Indianapolis, IN: Hackett Publishing Co. Ltd.

[bibr9-20427530221117329] Iliza A (2020) Internet Charges Waived for Students amidst COVID-19 Lockdown. Kigali, Butare: The New Times.

[bibr10-20427530221117329] International Council for Open and Distance Education (2020) The COVID-19 Pandemic: Ugandan Students opt for Online Studying. Available at: www.icde.org/icde-blog/2020/3/30/the-covid-19-pandemic-ugandan-students-opt-for-online-studying (accessed 20 August).

[bibr11-20427530221117329] January J Madhombiro M Chipamaunga S, et al. (2018) Prevalence of depression and anxiety among undergraduate university students in low- and middle-income countries: A systematic review protocol. Systematic Reviews 7(1): 1–5. 29636088 10.1186/s13643-018-0723-8 PMC5894225

[bibr12-20427530221117329] Kirschner PA Karpinski AC (2010) Facebook® and academic performance. Computers in Human Behavior 26(6): 1237–1245.

[bibr13-20427530221117329] Leech NL Onwuegbuzie AJ (2007) An array of qualitative data analysis tools: A call for data analysis triangulation. School Psychology Quarterly 22(4): 557–584.

[bibr14-20427530221117329] Liang X (2004) Uganda Tertiary Education Sector Report. Washington, DC: World Bank

[bibr15-20427530221117329] Mabusela SM Adams JD (2017) Students’ experience of e-learning at a rural- university in South Africa. Gender and Behaviour 15(4): 10221–10235.

[bibr16-20427530221117329] Mania K Janse van Rensburg A Bird R, et al. (2017) Writing into design: An embedded writing course for architectural studies. South African Journal of Higher Education 31(5): 172–188.

[bibr17-20427530221117329] Melles G (2008) Subscribed content producing fact, affect and identity in architecture critiques a discourse analysis of student and faculty discourse interaction. Art, Design & Communication in Higher Education 6(3): 159–171.

[bibr18-20427530221117329] Morkel J (2011) Facebook-enhanced face to face learning: The architecture studio. In: Genç Z (ed), 5th International Computer & Instructional Technologies Symposium. Elazığ, Turkey: Fırat University, pp. 346–352.

[bibr19-20427530221117329] Nowell LS Norris JM White DE, et al. (2017) Thematic analysis: Striving to meet the trustworthiness criteria. International Journal of Qualitative Methods 16(1): 1–13.

[bibr20-20427530221117329] Nsibirano R Kabonesa C (2013) Factors Influencing the Uptake of Technology for Teaching, Learning and Assessment at Makerere University. Kampala: Makerere University.

[bibr21-20427530221117329] Ovuga E Boardman J Wasserman D, et al. (2006) Undergraduate student mental health at Makerere University, Uganda. World Psychiatry 5(1): 51–52. 16757997 PMC1472270

[bibr22-20427530221117329] Prensky M (2001) Digital natives, digital immigrants: Part 1. On The Horizon 9(5): 1–6.

[bibr23-20427530221117329] Shulman LS (2005) Signature pedagogies in the professions. Daedalus 134(3): 52–59.

[bibr24-20427530221117329] Tamale S (2020) Decolonization and Afro-Feminism. Ottawa: Daraja Press.

[bibr25-20427530221117329] Tshabalala M Ndeya-Ndereya C van der Merwe T (2014) Implementing blended learning at a developing university: Obstacles in the way. Electronic Journal of E-Learning 12(1): 101–110.

[bibr26-20427530221117329] Tumusiime H (2013) Learning in architecture: Student's perceptions of the architecture studio. In: Farrow V (ed), Association of Architectural Educators (AAE) Inaugural Conference. Nottingham, UK: Nottingham Trent University.

[bibr27-20427530221117329] Van Reijswoud V Mulo E (2006) Applying open source software in a development context: Expectations and experiences. A Case Study of a University in Uganda E-Learning and Digital Media 3(3): 361–372.

[bibr28-20427530221117329] Wanner GK Phillips AW Papanagnou D, et al. (2019) Assessing the use of social media in physician assistant education. International Journal of Medical Education 10: 23–28. 30694797 10.5116/ijme.5c14.ef82 PMC6387779

[bibr101-20427530221117329] Olweny M (2017) Socialisation in architectural education: A view from East Africa. Education + Training 59(2): 188–200.

[bibr102-20427530221117329] Olweny M Morkel J Delport H, et al. (2021) Zombies in the Studio: Towards nurturing pedagogical approaches for architectural education in sub-Saharan Africa. Charrette 7(2): 57–83.

[bibr103-20427530221117329] Slee B Headley D De la Cruz E (2016) Sketching with someone 1000 Miles Away: A Report on the First Trials. In: Sustainable Futures Conference (SFC2016) - Architecture and Construction in the Global South (eds Tuts R and Gampfer S), Nairobi, Kenya. Stellenbosch University.

